# Health Behaviors as a Mediator of the Association Between Interpersonal Relationships and Physical Health in a Workplace Context

**DOI:** 10.3390/ijerph16132392

**Published:** 2019-07-05

**Authors:** Maria Alzira Pimenta Dinis, Helder Fernando Pedrosa Sousa, Andreia de Moura, Lilian M. F. Viterbo, Ricardo J. Pinto

**Affiliations:** 1 UFP Energy, Environment and Health Research Unit (FP-ENAS), University Fernando Pessoa (UFP), Praça 9 de Abril 349, 4249-004 Porto, Portugal; 2 Department of Mathematics (DM. UTAD), University of Trás-os-Montes and Alto Douro, Quinta de Prados, 5001-801 Vila Real, Portugal; 3 Digital Human-Environment Interaction Lab (HEI-Lab), Lusófona University of Porto (ULP), Rua Augusto Rosa 24, 4000-098 Porto, Portugal

**Keywords:** physical health, health behaviors, interpersonal relationships, mediator, predictor

## Abstract

The etiology of diseases is multifactorial, involving genetic, environmental, and lifestyle-related behaviors. Considering the pathway that involves behavioral processes, a huge body of empirical evidence has shown that some healthy behaviors such as non-smoking, any or moderate alcohol consumption, a healthy diet, (e.g., fruit and vegetable intake), and physical activity, decrease the risk of disease and mortality. This study aimed to explore the potential mediating effect of combined health behaviors on the association between interpersonal relationships and physical health in a Brazilian adult worker population from the Occupational Health Service within the oil industry in Bahia, Brazil. The sample included 611 workers, of which 567 (92.8%) were males and 44 (7.2%) females, age ranging from 18 to 73 years (*M* = 41.95; *SD* = 8.88). The significant predictors of physical health were interpersonal relationships and health behaviors. Health behaviors contributed significantly to a reduction in the effect of interpersonal relationships on physical health outcomes. As far as it is known, there has been no prior work in Brazil that simultaneously examined the best predictors of physical health in oil workers using this conceptual model. Interventions in the workplace environment need to consider health behavior as a mediator between interpersonal relationships and physical health, aligned in a global psychosocial approach to health at work.

## 1. Introduction

One of the most important goals of medicine and public health is to prevent disease. The etiology of diseases is multifactorial, involving genetic, environmental, and lifestyle-related behaviors. Considering the pathway that involves behavioral processes, a huge body of empirical evidence has shown that some healthy behaviors such as non-smoking, any or moderate alcohol consumption, a healthy diet (e.g., fruit and vegetable intake), and physical activity decrease the risk of disease and mortality [1,2,3,4,5,6,7,8,9,10,11,12,13]. 

The individuals’ social relationships have been linked not only to mental health but also to morbidity and mortality [14,15]. The two general theoretical models that propose processes through which social relationships may influence health are the stress buffering and main effects models. The buffering hypothesis [16] suggests that social relationships may provide resources (e.g., informational, emotional, or tangible) that promote adaptive behavioral or neuroendocrine responses to acute or chronic stressors and thus moderate or buffer the deleterious influence of stressors on health. The main effects model [17] proposes that social relationships may be associated with social influences, services, information, and psychological states that may influence engagement with healthy behaviors, which in turn influence the biological systems (e.g., endocrine, immune), and lastly influence physical health. According to some theorists, such as Umberson [18], Lewis and Rook [19], these health behaviors may mediate the connections between social relationships and health outcomes that are presented in the literature [14,17,20,21,22,23,24]. For example, perceived social support has been linked to better health behaviors, such as fruit and vegetable consumption and exercise [25,26]. While perceived stress increases the probability for risk-taking behaviors [27], family, friends, and coworkers are likely to observe such behavioral changes and comment or attempt to intervene, such as pressing the subject to seek help [28]. Accordingly, social relationships facilitate healthier behaviors and adherence to medical regimens, which in turn protect the subject from developing the disease. Additionally, other potential mechanisms that might explain how social support can influence health are related to biological mechanisms, especially the immune-mediated inflammatory processes [29,30]. 

Despite evidence on health behavior mechanisms linking social support to physical health outcomes, the major models of social support and health have privileged psychological mechanisms such as perceived stress, depression, and positive effect [31]. Furthermore, much of the empirical evidence on the connection between lifestyle-related behaviors and physical health have been obtained from a single behavior and treat other health behaviors as confounds [32,33]. Studies on the relationship between physical activity and the prevention of coronary heart disease have included dietary factors such as covariates [7,34]. Furthermore, meta-analysis and systematic reviews have shown that the study of the relationship between health behaviors and disease and mortality usually do not include social relationships (e.g., Loef and Walach [5]). Finally, regardless of the three decades of studies that have verified the beneficial effects of social ties on health, much empirical evidence is needed, with special attention to different cultural contexts, where seeking support, social norms, and independence versus interdependence in the context of social relationships, differ [28]. 

Therefore, given the scarcity of international studies on this matter, specifically in Brazil, and considering the previous arguments, this study intends to explore the potential mediation effect of combined health behaviors on the connection between interpersonal relationships and physical health in a Brazilian adult worker population within the oil industry. Additionally, the correlations among variables, including sociodemographic ones, were explored, and the independent contribution of each variable in accounting for physical health outcomes, after adjusted for covariates (e.g., occupational stressors), were examined.

## 2. Methods

### 2.1. Participants

The sample included 611 workers, of which 567 (92.8%) were males and 44 (7.2%) were females, with an age range of 18 to 73 years (*M* = 41.95; *SD* = 8.88), all of whom were recruited from the Occupational Health Service of the Brazilian oil industry in Bahia. The exclusion criteria included participants with cognitive limitations or psychiatric disorders, as they do not allow the correct accomplishment of the electronic medical records. The inclusion criteria referred to patients (i.e., workers) from the Occupational Health Service of the Brazilian oil industry in Bahia.

The questionnaires were applied at the facilities of the mentioned Occupational Health Service, in a private, quiet room. The data were identified only with the registration numbers, thus ensuring confidentiality. The data were collected through electronic medical records for the year 2017 and the consultations were carried out throughout the year 2017. 

All the users of the occupational health service were invited to participate in the study in person at the time of consultation of medicine and/or nursing and, thus, the sample is a convenience sample. After verbal consent, the data were collected under the medical database under analysis. The evaluation protocol took an average of 45 minutes to complete.

The study was approved by the Occupational Health Service of the Brazilian oil industry in Bahia and was also approved by the Ethics Committee of the Bahian School of Medicine and Public Health, based on the opinion of the Research Ethics Committee, CAAE no. 84318218.2.0000.5544, Brazil. All aspects related to privacy, confidentiality, access, and use of medical records, in the sense of compliance with ethical and legal requirements, were taken into care. In line with the guidelines of the Federal Medical Council, Brazil, the institution’s corporate guidelines state that only the Coordinator of the Occupational Health Medical Control Program may authorize access to medical and administrative information recorded in medical records. Accordingly, this study was officially authorized and signed by the responsible authority.

This evaluation clearly had a double function: i) a medical evaluation that benefited the patients alone, that allowed to guide and optimize the medical intervention; and ii) participation in research. Assuming these premises, patients participating in this study were given an opportunity to improve or deepen their medical assessment in several dimensions, the variables under analysis, and thus, also ensure adequate and close intervention to the needs identified in a personalized way. This was a reward which was verbalized by the researchers as felt by the participants, considering the subsequent interventions based on the results of the extensive medical evaluation.

### 2.2. Instruments

Sociodemographic Questionnaire. A single worksheet to collect information on gender, age, education, birthplace, civil status, and nationality, was used.

Electronic Medical Records of the Year 2017. Software for collecting data on physical health, nutrients intake, health behaviors, interpersonal relationships, and workplace environment, was used. The individuals were evaluated by doctors, psychologists, nurses, and other health professionals in terms of the degree to which each problem affected them, on a Likert-type scale (i.e., 0 to 5: 0—absence to 5—high presence/frequency). Several items were grouped into outcome variables, as indicated below. The collection of electronic medical records was approved by the Directorate of the Occupational Health Service and the Ethics Committee of the Bahian School of Medicine and Public Health, based on the opinion of the Research Ethics Committee, CAAE no. 84318218.2.0000.5544, Brazil, as a valid instrument for the clinical evaluation of the composite variables under analysis: physical health, nutrients intake, combined health behaviors, interpersonal relationships, and workplace environment. Lower scores mean lower physical health, poor nutrients intake, lower combined health behaviors, and lower quality of interpersonal relationships. Conversely, lower scores may mean a better workplace environment.

### 2.3. Outcome Variables

Physical Health. This composite variable includes different dimensions, namely: Classification of flexibility, abdominal strength, strength of arms, oral hygiene, pain when exercising physical activity, diabetes melitus, glycemia, hypertension, blood pressure, dyslipidemia, body weight, periodontal community index, and personal characteristics. The internal consistency was 0.71 and the score ranged from 0 to 65. 

Nutrients Intake. This composite variable includes different dimensions, namely: Simple carbohydrate, fibers, saturated lipids, mineral sodium, and liquids. The internal consistency was 0.61 and the score ranged from 0 to 25. 

Health Behaviors. This composite variable includes different dimensions, namely: Physical activity level, smoking, alcohol consumption, self-care level, food choices (fruit and vegetable intake), and oral hygiene. The internal consistency was 0.65 and the score ranged from 0 to 30.

Interpersonal Relationships. This composite variable includes different dimensions, namely: Socio-environmental components, family relations, social characteristics, and relationships in the workplace environment. The internal consistency was 0.60 and the score ranged from 0 to 20.

Workplace Environment. This composite variable includes different dimensions, namely: Exposure to risk agents, health surveillance, food safety, and environmental components. The internal consistency was 0.55 and the score ranged from 0 to 20. 

### 2.4. Data Analysis

Data analyses were carried out using the SPSS version 24 for Windows (IBM Corporation, New York, NY, USA). Descriptive statistics were calculated to characterize the study variables. The Pearson correlation test was used to examine the associations among the study variables. Hierarchical multiple regression analysis was used to calculate the independent contribution of workplace environment, nutrients intake, interpersonal relationships, and health behaviors in order to provide an estimate of incremental variance accounting for physical health, after being adjusted for age and gender. The mediation analyses were carried out with the PROCESS model [35] to SPSS. The bootstrapping technique with estimated coefficients from 5000 bootstrap samples was applied to determine direct and indirect effects. Confidence intervals (*CI*) that do not contain zero indicate a significant indirect effect. Pairwise deletion was used to handle missing data.

## 3. Results

### 3.1. Descriptive

Descriptive statistics of key measures are presented in Table 1. The total score was used for all variables.

### 3.2. Correlations

The correlations among the key measures are presented in Table 2. Physical health was significantly associated with all key measures, except with the workplace environment.

### 3.3. Hierarchical Multiple Regression Analysis to Predict Physical Health

The results from the hierarchical regression analyses are presented in Table 3. The first block, including age and gender, significantly contributed to the regression model, *R^2^* = 0.06, *F*(2, 565) = 19.69, *p* < 0.001, and Cohen’s *f*^2^ = 0.06. Adding the workplace environment in the second block, the model remained statistically significant, *F*(3, 564) = 13.17, *p* < 0.001, but this variable did not significantly contribute to the explained variance. Nutrients intake and interpersonal relationships were entered in the third step, and the model remained significant, *F*(5, 562) = 32.84, *p* < 0.001, and Cohen’s *f*^2^ = 0.21, contributing an additional 16% to the explained variance. Health behaviors were added in the final step, and the model remained significant, *F*(6, 561) = 82.22, *p* < 0.001, and Cohen’s *f*^2^ = 0.44, contributing an additional 24% to the explained variance. The final model explained 47% of the variance, with Cohen’s *f*^2^ = 0.89. 

### 3.4. Mediation Analysis

Simple mediation analyses, the most commonly employed type of mediation model, were conducted, cf. model 4 in Hayes [35]. The interpersonal relationships variable was input into the model as predictor, health behaviors as mediator, and physical health as outcome variable. A statistically significant effect of the interpersonal relationships on health behaviors (*B* = 0.54, *SE* = 0.06, *p* < 0.001), was observed. The total effect of the interpersonal relationships on physical health outcomes was significant (*B* = 1.06, *SE* = 0.14, *p* < 0.001), but their effect was significantly reduced (*B* = 0.30, *SE* = 0.12, *p* < 0.01) when health behaviors was controlled (*B* = 1.41, *SE* = 0.08, *p* < 0.001). The analysis of the indirect effect with bootstrapping data extracted, supported a significant level (*B* = 0.76, *SE* = 0.10, 95% *CI* (0.58, 0.96). 

## 4. Discussion

The current study aimed to explore the potential mediation effect of combined health behaviors on the connection between interpersonal relationships and physical health in a Brazilian adult workers population in the oil industry. 

The findings confirmed expectations that health behaviors mediated the relationship between interpersonal relationships and physical health in the sample. Several evidences have shown the link between social relationships and health outcomes [14,17,21,23,24], but most of the mechanisms that have been studied are perceived stress, depression, and positive affect [31], which affect the physical health through the immune system and inflammatory processes [29,30,36,37,38,39,40]. Besides the stress buffering and main effects models to explain the relationship between interpersonal relationships and physical health, the findings suggest a mediational model since the impact on health was also driven on behaviors, as smoking, alcohol consumption, a poor diet, and lack of physical activity, relevant in the field of worker’s health [41]. 

While the link between interpersonal relationships and physical health is well known in the literature (e.g., Feeney and Collins [42]), theoretically driven on stress buffering and main effects models, less information exists whether health behaviors work as mediational mechanisms on this relationship. On one hand, the engagement in health-risk behaviors act as compensatory factors to cope with interpersonal problems or isolation. According to some theoretical models (e.g., self-medication hypothesis and experiential avoidance model) [43,44], some behaviors are taken to avoid or escape from unwanted emotional states, such as shame, sadness, frustration, or reducing interpersonal conflicts [43]. This hypothesis is not new in the field of adverse childhood experiences. Several years ago, the CDC-Kaiser Permanente Adverse Childhood Experiences (ACE) study [45] hypothesized that the relationship between exposure to adversity and premature death was explained by behaviors such as smoking, alcohol or drug abuse, overeating, or sexual behaviors [46]. The authors of that study argued that individuals exposed to adversity could consciously or unconsciously use such behaviors, considering their pharmacological or psychological benefit, as coping devices in the face of the stress of abuse, domestic violence, or other forms of family and household dysfunction. On the other hand, the interpersonal relationships may provide incentives for engagement in health behaviors and thereby protect people from disease. As previously mentioned, interpersonal relationships could facilitate healthier behaviors, such as non-smoking, any or moderate alcohol consumption, a healthy diet (e.g., fruit and vegetable intake), and physical activity, which in turn protect the subject from developing the disease.

## 5. Conclusions

As far as it is known, there has been no prior work in Brazil that simultaneously examined the best predictors of physical health in oil workers using this conceptual model. Specifically, the results from this study suggest that nutrients intake, interpersonal relationships, and health behaviors are predictors of physical health.

In this sense, interventions in the workplace environment and, specifically, interventions in health at work need to focus on these factors, aligned with a psychosocial approach to this phenomenon. It is therefore important that planning interventions and regular monitoring of workers in their workplaces should consider this psychosocial and holistic approach. Finally, these results acknowledge the success of health interventions in this particular industry under study.

Furthermore, the findings added to the theory that the possible existence of a mediational model where health behaviors have an important role as a mediator between interpersonal relationships and physical health. Additionally, in terms of practical implications, the findings of this study make a compelling case for interpersonal relationships to be recognized by companies as a risk factor for engaging in risk-behaviors and consequently the development of disease. With such recognition, the occupational health services need to include the assessment of quality of interpersonal relationships of their workers, both at work and in a family context.

Conducted in SPSS with the PROCESS model, with a theoretical background compatible with the statistical analyses carried out, and based on the recent literature on this subject, this preliminary study presents statistically significant and theoretically relevant results, allowing researchers to guide studies towards an increasingly robust and up-to-date mediation effects and processes.

## 6. Limitations and Future Directions

This study has two main limitations that can be addressed in future research.

First, the sample is not representative of the population of oil workers in Brazil, so care must be taken when it comes to generalizing the results. However, specifically, the sample is representative of the population of workers recruited from an Occupational Health Service within the Brazilian oil industry in Bahia.

Additionally, a mediational hypothesis implies a causal sequence that cannot be tested with cross-sectional data. Considering that this is a cross-sectional study, any causality relationships between the variables under study must be interpreted with caution. Nevertheless, the psychopathology symptoms or other psychological diseases at the moment of the questionnaire being administrated, were not accessed. Finally, being a cross-sectional study, any causality relationships between the variables under study must be interpreted with care. In spite of the cross-sectional study design, these results point to the success of independent variables, i.e., nutrients intake, interpersonal relationships and health behaviors, in predicting the dependent variable, physical health.

Future studies should explore associations between mechanisms, such as relationships-related health behaviors, and other competing mechanisms, such as depression and anxiety.

## Figures and Tables

**Table 1:** Descriptive statistics for the study variables (*N* = 571).

Variables	Total Sample					
	*M*		*SD*		*Min*	*Max*
Physical health	43	0.72	7	0.32	19	61
Workplace environment	12	0.19	1	0.23	7	18
Nutrients intake	13	0.59	1	0.62	7	22
Interpersonal relationships	13	0.35	2	0.06	5	19
Health behaviors	20	0.04	3	0.34	7	30

*M*: mean; *SD*: standard deviation; *Min*: minimum; *Max*: maximum.

**Table 2:** Correlations for the study variables.

Variables	1	2	3	4	5
1. Physical health	-				
2. Workplace environment	−0.01	-			
3. Nutrients intake	0.28***	0.13**	-		
4. Interpersonal relationships	0.32***	0.18***	0.09	-	
5. Health behaviors	0.65***	0.03	0.39***	0.37***	-

**p < 0.01, two-tailed; ***p < 0.001.

**Table 3:** Hierarchical regression analyses predicting physical health.

Model^a^	*B*	*β*	*t*
Step 1: *R^2^* = 0.06**			
Age	−0.14	−0.18	−4.27***
Gender	4.73	0.16	3.90***
Step 2: *R^2^* = 0.06; Δ*R*^2^ = 0.00			
Workplace environment	−0.10	−0.02	−0.43
Step 3: *R^2^* = 0.23***; Δ*R*^2^ = 0.16***			
Nutrients intake	1.00	0.22	5.75***
Interpersonal relationships	1.11	0.32	8.35***
Step 4: *R^2^* = 0.47***; Δ*R*^2^ = 0.24***			
Constant	17.91		
Age	−0.12	−0.15	−4.86***
Gender	2.93	0.10	3.14**
Workplace environment	−0.28	−0.05	−1.52
Nutrients intake	0.08	−0.06	0.50
Interpersonal relationships	0.43	0.12	3.64***
Health behaviors	1.33	0.58	15.97***

^a^ Only Steps 1 and 4 show the complete model for that step. Other steps show only new variables, and coefficients are not for interpretation. ** *p* < 0.01; *** *p* < 0.001, two-tailed.
